# EHMTI-0097. Long-term outcome in 201 patients with chronic headache with medication overuse

**DOI:** 10.1186/1129-2377-15-S1-D78

**Published:** 2014-09-18

**Authors:** J Zidverc-Trajkovic, T Pekmezovic, A Radojicic, A Podgorac, N Sternic

**Affiliations:** 1 Headache Center, Neurology Clinic, Belgrade, Serbia; 2 Institute of Epidemiology, Faculty of Medicine University of Belgrade, Belgrade, Serbia; 3 Headache Centar, Neurology Clinic, Belgrade, Serbia; 4 Neurology, Faculty of Medicine University of Belgrade, Belgrade, Serbia

## 

From the cohort of 240 patients with chronic headache with medication overuse (MOH), treated with drug withdrawal and prophylactic medications and evaluated at 1-year follow-up, 57.1% were without chronic headache and without medication overuse, 3.3% did not improve after drug withdrawal and 39.6% relapsed developing recurrent overuse (Cephalalgia 2007; 27:1219-25).

The aim of the present study was to evaluate the long-term outcome of these patients.

During the next 1-12 years, follow-up examinations were performed in 201 (83.8%) patients. There were no significant differences between patients lost for further examination and other patients regarding age and gender, as well as the outcome on 1-year follow-up.

On the last follow-up, 66 (32.8%) patients had chronic headache with medication overuse. Without overuse were 130 (64.7%) patients with episodic and five (2.5%) patients with chronic headaches. During the follow-up period, 47 (23.4%) patients had relapsed developing recurrent overuse. The recurrent overuses occurred once in 33 (16.4%), twice in 13 (5.0%) and thrice in four (2.0%) patients. MOH recurrence occurred during the first three years after the first-year follow-up in three quarters of patients. The majority of patients, 33 (70.2%), overused the same medication. Treatment of MOH recurrence was efficacious in 93.6% patients, with strong advice to cease overused drug in 79.0% and prophylactics in 83.0% patients. During the examined period 20 (23.3%) of the patients with MOH on the first-year follow-up had remission of chronic headache with subsequent decrease of medication use.

No conflict of interest.

